# The Role of Hydroxyl Modification of Peptidoglycan to Reduce the TTX Toxicity via Superior Absorption

**DOI:** 10.3390/foods14122145

**Published:** 2025-06-19

**Authors:** Chang’e Wang, Yi Guo, Lili Zhang, Junjian Miao, Ying Lu

**Affiliations:** 1 College of Food Science and Technology, Shanghai Ocean University, Shanghai 201306, China; 2 Shanghai Engineering Research Center of Aquatic-Product Processing and Preservation, Shanghai 201306, China; 3 National R&D Branch Center for Freshwater Aquatic Products Processing Technology (Shanghai), Shanghai 201306, China; 4 Marine Biomedical Science and Technology Innovation Platform of Lingang Special Area, Shanghai 201306, China

**Keywords:** hydroxyl modification of peptidoglycan, tetrodotoxin toxicity, adsorption capacity, physicochemical interaction, electrostatic interaction

## Abstract

The by-products that may contain tetrodotoxin (TTX) produced during the processing of farmed pufferfish have caused food safety risks and environmental pollution. Peptidoglycan (PG) of lactic acid bacteria could adsorb TTX; however, its complex structure and poor solubility limited adsorption efficiency. In this study, hydroxyl modifications of three PGs (A3α, A1γ and A4α) were realized via TEMPO-mediated selective oxidation of the primary hydroxyl group. Compared with PGs, it was found that the carboxyl density of hydroxyl-modified PGs (HM-PGs) increased from 1.66 mmol/g to 3.33 mmol/g and the surface electronegativity increased from −36 mV to −59 mV. The adsorption capacity of HM-PGs to TTX reached 1.48 μg/mg, which was comparable to the adsorption of the conventional adsorbent chitosan for aflatoxin B1 (1.39 μg/mg). Moreover, HM-PGs decreased the toxicity of TTX from strong toxic to nearly non-toxic, with the toxicity reduction rate reached 99.85%. After treatment with HM-PGs, the mouse hippocampus and neuronal cell model confirmed that lower neural injury and sodium channel blocking effects were observed in the residual TTX, whose neurotoxicity was lower. Molecular docking simulation and physicochemical analysis revealed that the adsorption of TTX by HM-PGs was a complex adsorption mode driven by the synergy of physicochemical interaction. There were both physical adsorptions based on electrostatic and hydrophobic interactions and chemical binding with strong hydrogen bonding (1.46 Å) and Mayer bond order (0.1229). This study not only developed a new, efficient and safe tool for TTX removal, but also provided a theoretical basis for the development of biological toxin removal material.

## 1. Introduction

Pufferfish is regarded as a high-value aquatic commodity with increasing demand in East Asian countries, particularly Japan and China, due to its unique flavor and high-quality protein content [1]. However, tetrodotoxin (TTX), a potent neurotoxin found in pufferfish, can cause nerve paralysis, respiratory failure and even death by blocking voltage-gated sodium channels (VGSCs) [2]. The advances in selective breeding and standardized aquaculture have enabled the low-toxicity pufferfish culturing industry to develop rapidly. Ji et al. [3] reported that high concentrations of tetrodotoxin (TTX, up to 125 mouse units per gram (MU/g)) might still accumulate in non-muscle tissues such as the liver and ovaries, posing a food safety risk. In Japan, a strict regulatory limit of TTX (equivalent per kg) has been established for food safety [4]. Based on safety considerations, the current standard in China only allowed the muscle of cultured pufferfish to be marketed [5], resulting in a large number of by-products containing TTX being discarded. This issue led to resource waste and environmental risk. Therefore, developing effective strategies to remove or neutralize TTX of by-products has become an urgent priority.

Traditional TTX removal strategies, including thermal degradation [6], chemical alkalization [7] and microbial fermentation [8], were often hindered by complex operation protocols, low removal efficiency and potential generation of toxic by-products. In contrast, adsorption methods have become a research hot topic due to operational simplicity, mild reaction conditions and no by-product formation. In recent years, novel synthetic adsorbents, such as cyclodextrin polymers [9] and nerve cell membrane nanosponges [10], have been developed to target TTX removal. However, synthetic adsorbents face biosafety controversy and high costs, which limit industrial application.

As a result, natural polymer adsorbents such as chitosan, alginate and cellulose have increased attention due to renewability, excellent biocompatibility and environmental friendliness [11]. These adsorbents have rich reactive groups (amino, hydroxyl, carboxyl and sulfhydryl groups), which can be modified to enhance their adsorption efficiency and selectivity for toxins. For example, the removal of Ochratoxin A (OTA) from red wine by chitosan was 24.70% [12], which increased to 46.00% after magnetic modification [13], probably due to enhanced hydrogen bonding, electrostatic interactions and complexation between chitosan and OTA. Similarly, the introduction of quaternary ammonium and alkyl groups into the chitosan enhanced the electrostatic interaction with negatively charged toxins, increasing the removal efficiency of microcystins to 93.47% [14]. Liang et al. found that siliconization modification of cellulose gel membrane increased surface sulfhydryl content, enhancing patulin adsorption in fruit juice by 125-fold, which reached 498.78 μg/g [15]. Furthermore, the binding capacity of natural cellulose for Cu(II) was increased 2–3 fold after the introduction of carboxyl groups by carboxymethylation treatment [16]. These findings suggested that the introduction of functional groups, directional modification or grafting of natural polymers such as carboxyl, amino and sulfhydryl groups can significantly improve the adsorption performance of biotoxin. However, most studies focus on the evaluation of its adsorption capacity for toxins, and there are few studies on the toxicity-reducing effect of the residue after adsorption and its action mechanism.

Peptidoglycan (PG), a natural polysaccharide–peptide complex derived from lactic acid bacteria (LAB) has a unique three-dimensional mesh-like architecture and a high density of functional groups [17], demonstrating significant efficacy in biotoxin removal. For example, Tian et al. found that PG from LAB could remove 87.70% of aflatoxin B1 by adsorption [18]. Additionally, Sun et al. [19] found that PG could remove 75.13% of 2-amino-1-methyl-6-phenyl-imidazolium [4,5-b] pyridine by the binding action of its hydroxyl (O-H) and amino (N-H) groups. Our previous studies have confirmed that PG could remove TTX by forming hydrogen bonds, and electrostatic and hydrophobic interactions with guanidino and hydroxyl groups of TTX through amino, hydroxyl and carboxyl groups [20]. However, the complex structure and poor solubility of PG led to low adsorption efficiency. Therefore, improving the adsorption capability of PG has become a critical research focus in the future. Previous studies found that the chemical modification of hydroxyl and carboxyl groups could significantly enhance the adsorption capacity of natural polysaccharide polymers. For example, Gomez-Maldonado et al. [21] demonstrated that TEMPO-oxidized β-cyclodextrin could introduce carboxyl groups and significantly enhance the adsorption capacity for microcystin-LR from 2.36 mg/g to 20.50 mg/g. These findings indicated that the introduction of carboxyl groups was effective for the adsorption of heavy metals and toxins. Notably, PG contained the active functional groups such as hydroxyl and carboxyl groups have a good modification potential [22]. Therefore, improving the adsorption performance of PG to TTX through chemical modification might be a feasible solution.

**Scheme 1:**
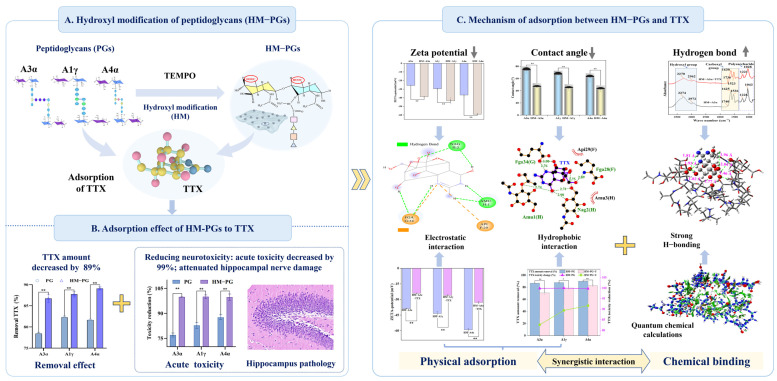
Establishment of the hydroxyl modification method of peptidoglycan (PG) and analysis of its adsorption effect on tetrodotoxin (TTX). Hydroxyl modification of peptidoglycans (HM-PGs) (**A**), adsorption effect of HM-PGs to TTX (**B**) and mechanism of adsorption between HM-PGs and TTX (**C**).  * indicate a statistically significant difference (*p* < 0.05); ** indicate an extremely significant difference (*p* < 0.01).

Currently, there are few studies on the modification of PG, most of the studies focused on the modification of LAB and its cell wall. For example, the esterification reaction increased the sulfhydryl content of *Lactobacillus plantarum* and removed over 90% of OTA [23]. As the main skeletal component of the LAB cell wall, PG provided higher structural flexibility for hydroxyl or carboxyl modification. Therefore, directional functional group modification of PG not only ensures biocompatibility and application safety, but may enhance its adsorption performance. In this study, three types of PGs (A3α, A1γ and A4α) were employed for hydroxyl modification by TEMPO/NaBr/NaClO. Firstly, the hydroxyl modification method was established through optimization, and the structural and physicochemical properties of hydroxyl-modified PGs (HM-PGs) were characterized by various spectroscopic and analytical techniques. Next, the TTX adsorption capacity of HM-PGs was evaluated by HPLC, and the neurotoxicity of residual was analyzed by mouse bioassay, mouse hippocampal injury model and neuronal cell model, respectively. Finally, the adsorption interaction between HM-PGs and TTX was predicted by molecular docking simulations, and validated by experimental approaches. This study developed an efficient and safe TTX adsorbent, which is expected to provide a theoretical basis for the development of biological toxin removal materials (Scheme 1).

## 2. Materials and Methods

### 2.1. Materials

LABs including *Enterococcus faecalis, Lactobacillus plantarum N115* and *Lactobacillus coryniformis I3* were prepared in-house at our laboratory (Shanghai, China). TTX standard (HPLC grade, ≥99%), fluorescein isothiocyanate dye (FITC, ≥90%), Cyanine 3 (Cy3) labeled goat anti-mouse IgG (Cy3-GAM IgG,), 2,2,6,6-tetramethylpiperidine-1-oxyl (TEMPO, analytical reagent: AR), NaBr (AR), NaClO (AR) and Na_2_S_2_O_3_ (AR) were obtained from Shanghai Aladdin Biochemical Technology Co., Ltd. (Shanghai, China). Ethanol (AR), NaOH (AR), Na_2_CO_3_ (AR), NaHCO_3_ (AR), phosphate buffer saline (PBS, AR), HCl (AR), anti-quenching solution (AR), acetic acid (HPLC grade, ≥99%) and urea (AR) were purchased from Sinopharm Chemical Reagent Co., Ltd. (Shanghai, China). Specific pathogen-free (SPF) grade Kunming male mice were supplied by Shanghai JieSiJie Laboratory Animal Co., Ltd. (Shanghai, China) and randomly divided into groups (6 mice per group) which could freely feed and drink water. SH-SY5Y cell and EMEM F-12K culture medium were obtained from Taiyuan Rosetta Stone Biotech Co., Ltd. (Shanxi, China). SBFI AM (Na^+^ Indicator) was purchased from Shanghai Maokang Biotechnology Co., Ltd. (Shanghai, China). Perch samples were supplied by Jiangsu Zhongyang Group Co., Ltd. (Nantong, China). The monoclonal antibody (mAb) TTX was purchased from Wuxi Determine Biotechnology Co., Ltd. (Wuxi, China).

### 2.2. Preparation of Hydroxyl-Modified PG

The hydroxyl-modified PGs (HM-PGs) were prepared according to Wang et al. [24]. Briefly, 200 mg of PGs was mixed in 20 mL deionized water containing TEMPO (50 mg) and NaBr (50 mg) under constant stirring. The mixture was added NaClO drop by drop for oxidation while the pH was adjusted using 0.5 M NaOH to maintain at 8.5. The oxidation product was stirred at 4 °C for 60 min. The reaction was terminated with anhydrous ethanol, and residual oxidants were neutralized with Na_2_S_2_O_3_. After adding four volumes of ethanol and incubating overnight at 4 °C, the precipitate was collected by centrifugation, washed with 70% ethanol, dialyzed and lyophilized to obtain HM-PGs (HM-A3α, HM-A1γ, HM-A4α). Reaction parameters were optimized based on carboxyl content as the evaluation index, including NaClO concentration (ranging from 0 to 0.2 mM), pH (ranging from 7.0 to 10.5), temperature (ranging from 0 to 50 °C) and time (ranging from 10 to 120 min).

### 2.3. Determination of Carboxyl Group Content of PGs and HM-PGs

The carboxyl content of PGs and HM-PGs was determined by Boehm titration [25]. The samples were titrated with 0.5 M NaOH, Na_2_CO_3_ and NaHCO_3_ solutions, respectively, with 0.05 M HCl solution using methyl red as an indicator. The carboxyl content (mmol/g) was calculated according to the following formula (1)–(4), and the average of the three titrants was used as the final value.

(1) $$n_{\left(NaOH\right)}=\frac{C_{NaOH}V_{NaOH}-C_{HCl}V_{HCl}}{W}$$

(2) $$n_{{(Na}_{2}{CO}_{3})}=\frac{{2C}_{{Na}_{2}{CO}_{3}}V_{{Na}_{2}{CO}_{3}}-C_{HCl}V_{HCl}}{W}$$

(3) $$n_{\left(NaH{CO}_{3}\right)}=\frac{{2C}_{NaH{CO}_{3}}V_{NaH{CO}_{3}}-C_{HCl}V_{HCl}}{W}$$

(4) $$n_{\left(COOH\right)}=\frac{(nNaOH+n_{{Na}_{2}{CO}_{3}}+n_{NaH{CO}_{3}})\times 45.02}{3\times 1000}$$

where *n* represents the calculated carboxyl group content of NaOH, Na_2_CO_3_ and NaHCO_3_ (mmol/g); C represents the concentration of NaOH, Na_2_CO_3_ and NaHCO_3_ (0.5 M); *V* represents the volume of NaOH, Na_2_CO_3_ and NaHCO_3_; C(HCl) represents the concentration of HCl (0.05 M); *V(HCl)* represents the volume of HCl consumed during titration (mL); *W* represents the mass of the sample (g); and 45.02 represents the relative molecular mass of the carboxyl group (g/mol).

### 2.4. Structural and Property Characterization of HM-PGs

Scanning electron microscopy coupled to energy dispersive spectroscopy (SEM-EDS) analyses were performed by JEOL JSM-IT 300LV Scanning Electron Microscope (Tokyo, Japan) equipped with Oxford INCA Energy 200 EDS SATW detector (WD 10, KV 5; High Wycombe, UK). EDS data were processed with AZTEC software (version 5.0, Oxford Instruments). The samples were diluted to 100 μg/mL using 0.1 M phosphate buffer (pH 7.0), and 20 μL were dropped onto a clean mica sheet and dried at room temperature. The surface morphology of PGs and HM-PGs was observed by atomic force microscopy (AFM, Bruker Nano GmbH, Berlin, Germany), and analyzed using NanoScope Analysis 1.80 software. Fourier transform infrared spectroscopy (FTIR, Thermo Nicolet iS 10, Waltham, MA, USA) was recorded in absorbance mode (400–4000 cm^−1^, 4 cm^−1^ resolution, 32 scans) [26]. Particle size and Zeta potential were measured by dynamic light scattering Zetasizer (Malvern Zetasizer pro, Graz, Austria).

### 2.5. Fluorescence Imaging of HM-PGs Treated with TTX Samples

HM-PG samples were labeled with FITC dye. The HM-PG-TTX was sequentially labeled with mAb (50 ng/mL) and Cy3-GAM IgG (1:200, *v*/*v*). After the addition of an anti-quenching solution (50 μL), the HM-PG and HM-PG-TTX samples were observed by confocal laser scanning microscope (CLSM, TCS SP8, Leica Microsystems, Wetzlar, Germany), with excitation and emission wavelengths ranging from 492 nm to 520 nm for FITC (green fluorescence), and from 548 nm to 563 nm for Cy3 (red fluorescence), respectively.

### 2.6. Quantification and Toxicity Analysis of TTX Samples

The amount and toxicity of TTX were assessed using the HPLC and mouse bioassay method outlined in the National Standard of the People’s Republic of China (GB 5009.206-2016 [27]). Quantitative analysis of TTX was performed using an HPLC system (Waters e2695, Waters Corporation, Milford, MA, USA) equipped with a 2998 PDA detector at 200 nm, which corresponded to the maximum UV absorbance of TTX. Separation was achieved on a Diamonsil Plus C18-A column (5 μm, 4.6 × 250 mm, Dikma Technologies, Shanghai, China) at 30 °C. The mobile phase was 20% acetonitrile and 80% ultrapure water (*v*/*v*) at a flow rate of 0.6 mL/min, and the elution was carried out under isocratic conditions. The injection volume was 10 μL and the total running time was 15 min. All samples were filtered through a 0.22 μm syringe filter before injection. The TTX standard was dissolved in 0.1% acetic acid to prepare a stock solution of 1 mg/mL, and then diluted with ultrapure water to obtain the working standard solution. Standard curves were established using TTX concentrations of 1–50 μg/mL (y =14425x + 10504, *R*^2^ = 0.9996), and the TTX amount in each sample was calculated based on peak areas.

Animal experiments were approved by the Ethics Committee of Shanghai Ocean University (approval no. SHOU-DW-2021-003). A total of 66 SPF-grade male Kunming mice (4 weeks, 20 ±1 g) were randomly divided into 11 groups (n = 6), which could freely feed and drink water. The control group received an intraperitoneal injection of 1 mL sterile saline. TTX groups received 1 mL of TTX standard solution (range from 0.25 to 3.0 μg/mL) to generate a toxicity standard curve based on time to death (y = 0.17134x + 0.10786, *R*^2^= 0.9834). The 9 experimental groups were intraperitoneally injected with 1 mL of sample supernatants (PGs: A3α, A1γ and A4α; HM-PGs: HM-A3α, HM-A1γ and HM-A4α); HM-PGs treated with urea (HM-PG-U: HM-A3α-U, HM-A1γ-U and HM-A4α-U). The toxicity reduction effect of HM-PGs on TTX was evaluated using a mouse bioassay and hippocampal injury model. The time of injection and death of each mouse were recorded and death was judged by the cessation of breathing standard. TTX toxicity was calculated using the method described by Liu et al. [20]. After the mice stopped breathing, the brain tissue samples were collected and stained with hematoxylin–eosin for pathological analysis. The hippocampus tissues of mice that stopped breathing were collected for pathological analysis. The surviving mice were anesthetized with isoflurane, and their hippocampal tissues were collected before euthanasia.

### 2.7. Determination of Intracellular Na^+^ Concentration Based on SH-SY5Y Cell Model

The SH-SY5Y cells were seeded and cultured in 96-well plates (2 × 10^4^ cells/well, 100 μL/well) for 24 h. Then, the cells were treated with different concentrations of TTX standard solutions to screen the appropriate administration concentration. The experimental groups were as follows: the control group (excluding TTX treatment), the TTX standard solution treatment group, the supernatant treatment groups after TTX adsorption by PGs (A3α, A1γ and A4α) and HM-PGs (HM-A3α, HM-A1γ and HM-A4α). After 2 h of treatment, cells were washed twice with PBS and incubated with 100 μL of 10 μM SBFI AM (Na^+^ Indicator) probe for 2 h. Then, the cell culture medium was added and incubated for 60 min to ensure sufficient probe uptake. Subsequently, the cells were washed three times with a serum-free medium to reduce background fluorescence. Fluorescence intensity was measured using a multifunctional microplate reader at dual excitation wavelengths of 340 nm and 380 nm, with emission wavelengths of 500 nm (emission wavelength range: 450–550 nm). The intracellular Na^+^ concentration was calculated based on the ratio of the fluorescence signals collected at the dual excitation wavelengths.

### 2.8. Prediction of Action Mode Between HM-PG and TTX

The semi-flexible molecular docking analysis was conducted using Auto Dock Vina 4.0 software (Center for Computational Structural Biology, CCSB) [28]. The TTX structure was obtained from the PubChem database (CID: 11174599). The structure of HM-PG was constructed by GaussView using the 2MTZ (chain B-H) of Bacillus subtilis as a template [29]. Docking results were visualized and analyzed using PyMOL software (the PyMOL Molecular Graphics System, Version 2.8 Schrödinger, LLC.). The intermolecular forces were analyzed by Biovia Discovery Studio (DS, 2019 version, Accelrys Inc., San Diego, CA, USA) and LigPlot+ software (version 2.2.4, EMBL-EBI, Hinxton, UK) [30]. Molecular dynamics (MD) simulations and semi-empirical optimization calculations were performed via Xtb [31]. First, MD simulations were performed at the generalized force field (GFN-FF), where water was used as an implicit solvent to obtain the lowest energy HM-PG structure (step size = 1 fs for 1000 ps, with results saved every 100 fs). Then, quantum mechanical semi-empirical optimization was performed at the GFN2-xTB level [32]. The HM-PG structure was optimized at the B3LYP(GD3BJ)/6-31G** level by density-functional theory (DFT) with Gaussian 16 C.02 (Gaussian, Inc., Wallingford, CT, USA) [33]. Further, the graphical representation of variational mode decomposition (VMD) was employed to visualize the interaction network and hydrogen bond interaction between HM-PG and TTX.

### 2.9. Analysis of Hydrophobicity and Toxicity Changes of HM-PGs

HM-PG (5 mg/mL) and HM-PG-U (5 mg/mL) solutions were mixed with an equal volume of hexadecane, respectively, and vortexed for 5 min. After the solution was stratified at 25 °C, the absorbance of the lower layer liquid at 600 nm was measured (HM-PGs: OD_1_, HM-PG-U: OD_2_). The hydrophobicity of HM-PGs before and after urea treatment was calculated according to the formula of hydrophobicity (%) = (1 − OD_2_/OD_1_) × 100%. Finally, 3 mg of HM-PGs and HM-PG-U were reacted with 1 mL of TTX (5 µg/mL), respectively. The amount and toxicity of TTX in the supernatant were determined by methods detailed in Section 2.6.

### 2.10. Statistical Analysis

Three parallel experiments were conducted for each sample. All data are presented as means ± standard deviation (SD, n = 3). Statistical analysis was performed using one-way ANOVA followed by Tukey’s multiple comparisons test. The statistical analyses covered data from physicochemical characterization, quantification of residual TTX, and in vitro and in vivo toxicity evaluation. Significance levels were indicated as follows: ns, not significant (*p* ≥ 0.05); *, statistically significant difference (*p* < 0.05); **, statistically extremely significant difference (*p* < 0.01). The graphs were plotted using GraphPad (Version 9.4.1; San Diego, CA, USA, www.graphpad.com, accessed on 10 February 2025) and Origin (Version 2021, Northampton, MA, USA; www.originlab.com, accessed on 10 February 2025) software.

## 3. Results and Discussion

### 3.1. Condition Optimization of HM-PG Preparation

**Figure 1:**
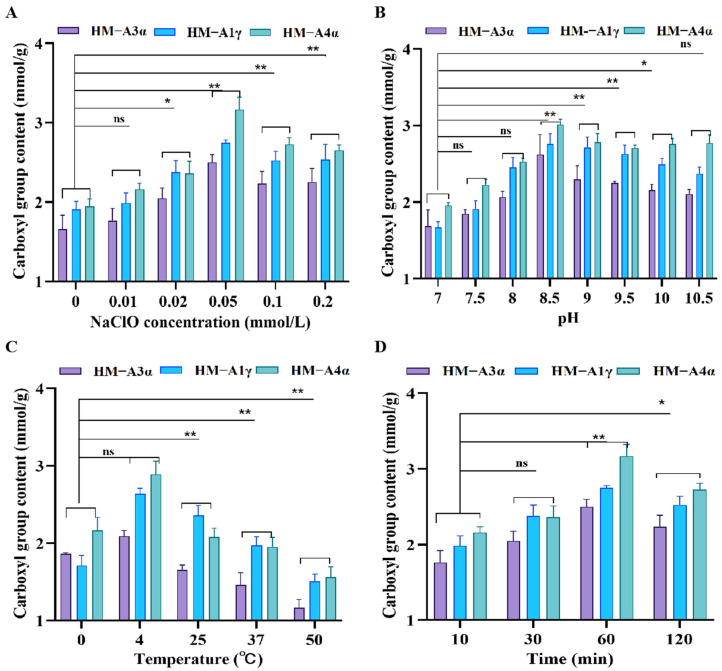
Effect of hydroxyl modification conditions on HM-PGs’ carboxyl contents. (**A**): NaClO concentration (mmol/L); (**B**): pH; (**C**): temperature (°C); (**D**): time (min). All data are expressed as mean ± SD (n = 3). Significance was analyzed within groups, ns indicates not significant (*p* ≥ 0.05), * indicates a statistically significant difference (*p* < 0.05); ** indicates a statistically extremely significant difference (*p* < 0.01).

It was previously demonstrated that the primary hydroxyl group of the PG sugar molecule plays a key role in TTX binding [20]. In this study, TEMPO was selected as a modifier due to its specific oxidation of the primary hydroxyl group to a carboxyl group, to explore the relationship between hydroxyl modification and TTX binding efficiency. Optimization of reaction conditions was conducted by varying NaClO concentration, pH, temperature and reaction time (Figure 1). The carboxyl content of three types of HM-PGs was initially increased and then decreased. The highest carboxyl content (2.4–3.3 mmol/g) was obtained under conditions of 0.05 mM NaClO (Figure 1A), pH 8.5 (Figure 1B), 4 °C (Figure 1C) and 60 min (*p* < 0.01, Figure 1D), which increased by 1.4–1.6 folds compared with unmodified PG. Wang et al. found that TEMPO-mediated oxidation modification increased the carboxyl content of cellulose/chitin, which was consistent with our results [24]. Notably, there was a difference in the degree of carboxyl group introduction among the three HM-PGs, which manifested as HM-A4α > HM-A1γ > HM-A3α, and this difference may derive from the initial structure of the PGs. The peptide bridge of HM-A4α consisted of aspartic acid (Asp) that possessed a native carboxyl side chain [34]. After TEMPO-oxidized the hydroxyl group, the carboxyl group was not only introduced to the sugar molecule, but the Asp side chain further increased the total carboxyl group content, which made the most significant modification effect. However, HM-A1γ was directly cross-linked through the peptide tail of diaminopimelic acid (Dap) [35], and the relatively open chain structure of Dap facilitated the diffusion of TEMPO, thus achieving a certain degree of oxidative modification, with a carboxyl group content among the three. In contrast, the peptide bridge of HM-A3α consisted mainly of alanine (Ala) residues and lacked a carboxyl side chain like that of Asp [17]. Therefore, the increase in carboxyl groups was almost entirely dependent on the oxidation of hydroxyl groups, which showed minimal carboxyl group content. In summary, the most efficient conversion of hydroxyl to carboxyl groups was achieved at 0.05 mM NaClO, 4 °C, pH 8.5 and 60 min, in which HM-A4α exhibited the greatest modification due to its sugar and peptide composition.

### 3.2. Effect of Hydroxyl Modification on Structure of Different PGs

**Figure 2:**
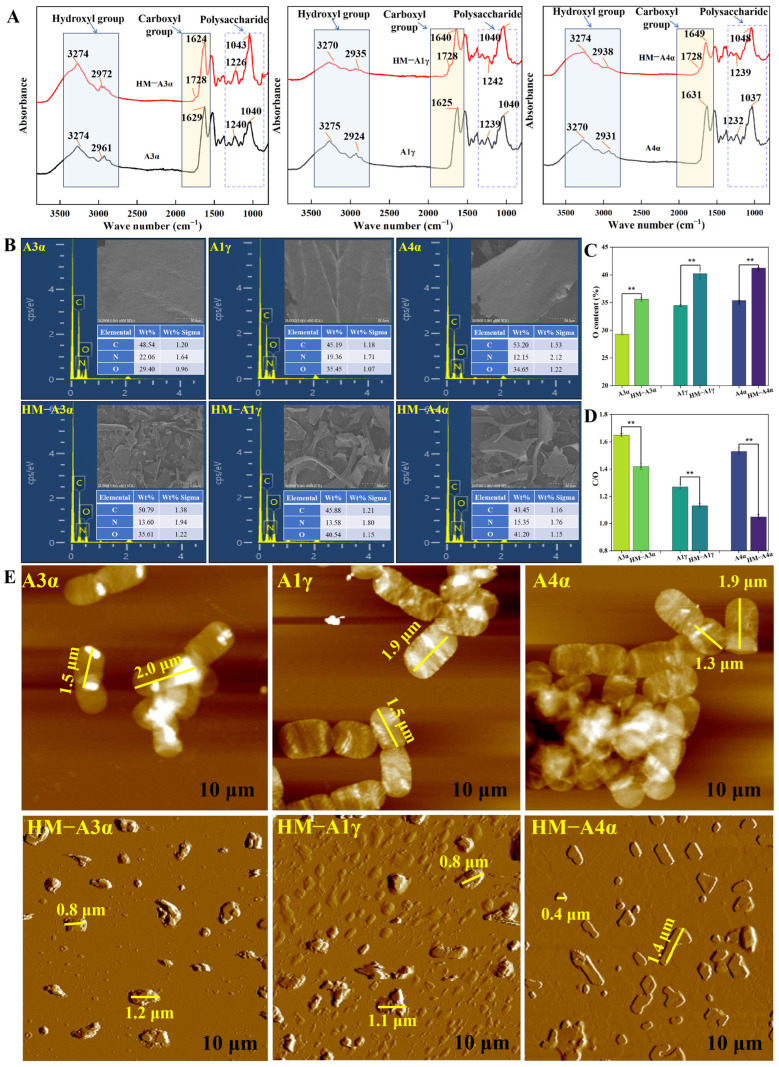
Analysis of the structure of three HM-PGs. The FTIR (**A**), SEM (**B**), O content (**C**) ratio C/O (**D**) and AFM (**E**) of PG before and after hydroxyl modification. All data are expressed as mean ± SD (n = 3). Significance between groups was analyzed; ** indicates a statistically significant difference (*p* < 0.01).

The structure changes of PGs before and after hydroxyl modification were characterized by FTIR. As shown in Figure 2A, the unmodified three PGs showed distinct O–H stretching vibration peaks at 2900–3300 cm^−1^, indicating the presence of hydroxyl groups. Characteristic polysaccharide backbone vibrations were observed in the regions 1200–1300 cm^−1^ and 1000–1100 cm^−1^, corresponding to asymmetric C–O–C and C––OH stretching, respectively [36]. Additionally, the adsorption between 1500 and 1700 cm^−1^ was attributed to the amide band, reflecting the glycopeptide structural features of PG. However, there were significant changes in the HM-PG spectra after hydroxyl modification. The most obvious alteration was the enhanced C=O stretching vibration at 1700–1750 cm^−1^, indicating the successful introduction of the –COOH group. Similarly, Sang et al. demonstrated that TEMPO treatment enhanced the C=O vibration at 1720 cm^−1^ in cellulose [36]. Furthermore, the hydroxyl stretching peaks in HM-A3α, HM-A1γ, and HM-A4α shifted from 3277 cm^−1^ to 3273 cm^−1^, 3275 cm^−1^ to 3270 cm^−1^ and 3270 cm^−1^ to 3265 cm^−1^, respectively, further confirming oxidation of hydroxyl groups occurred during the TEMPO modification process. Consequently, the TEMPO/NaBr/NaClO oxidation system successfully modified the hydroxyl group of PGs to the carboxyl group.

The effect of hydroxyl modification treatment on the microscopic morphology of PGs was investigated by SEM and AFM. SEM images showed that the surface morphology of PGs was smooth and flat, whereas the morphologies of the three HM-PGs were fragmented and broken (Figure 2B). It was probably due to the TEMPO treatment, which disrupted the original hydrogen bonding network of PG, and NaClO could induce oxidative rupture of β-1,4-glycosidic bonds, leading to depolymerization of the PG chains. Dasan et al. found that the TEMPO oxidation system could cause brittle failure of the surface structure of nanocrystalline cellulose, which was consistent with our research results [37]. EDS elemental analysis further confirmed chemical changes. Compared with the PGs, the oxygen content of the three modified HM-PGs increased significantly by 16.65% (*p* < 0.01, Figure 2C), and the C/O ratio decreased from 1.27 to 1.05 (*p* < 0.01, Figure 2D). This change was consistent with the increase in the content of carboxyl groups introduced during the hydroxyl modification process (Figure 1). Wang et al. also demonstrated that the TEMPO-mediated oxidation process significantly increased the carboxyl group content of cellulose and chitin, consistent with our findings [24]. In addition, the AFM images displayed that the PG had a skeleton morphology, and its surface showed an irregular, rough morphology with a length between 1.5 and 2.0 μm (Figure 2E). In contrast, three HM-PGs (HM-A3α, HM-A1γ and HM-A4α) lacked the backbone morphology, while surface roughness remained, particle sizes were notably reduced to 0.4–1.4 μm (Figure 2E). It suggested that the hydroxyl-modified treatment caused the degradation of PG, which was consistent with the results of SEM. In summary, the change of structure and morphology of HM-PGs proved that the TEMPO/NaBr/NaClO system successfully modified the hydroxyl group of PGs into the carboxyl group, and significantly changed the microscopic morphology and O elemental content of PGs.

### 3.3. Effect of Hydroxyl Modification on Physicochemical Properties of PGs

**Figure 3:**
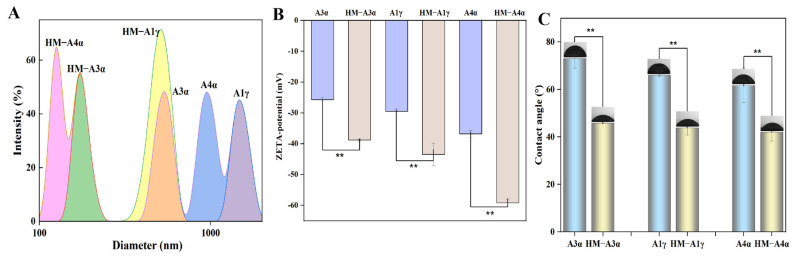
Analysis of the properties of three HM-PGs. The particle size (**A**), ZETA potential (**B**) and contact angle (**C**) of PG before and after hydroxyl modification. All data are expressed as mean ± SD (n = 3). Significance between groups was analyzed; ** indicates a statistically extremely significant difference (*p* < 0.01).

The effect of hydroxyl modification on the physicochemical properties of PGs was evaluated by measuring particle size, potential and contact angle. A decrease in the particle size of three HM-PGs was observed in Figure 3A. Jia et al. demonstrated that TEMPO-mediated oxidation could reduce the particle size of bacterial cellulose by partially disrupting inter- and intrafibrillar hydrogen bonds [38]. Therefore, we speculated that TEMPO oxidative might cause partial degradation of PGs. Zeta potential analysis indicated that the surface electronegativity of three HM-PGs was significantly higher than that of the unmodified PGs (*p* < 0.01, Figure 3B), which might be due to the introduction of negatively charged carboxyl groups. Similarly, Jia et al. found that the enhanced surface electronegativity of chitin nanocrystals after TEMPO oxidation was due to the successful introduction of carboxyl groups [39]. Among the three HM-PGs, HM-A4α exhibited the most negative Zeta potential (−59 mV), followed by HM-A1γ (−43 mV) and HM-A3α (−38 mV). This variation might be due to the fact that HM-A4α contained Asp peptide bridges carrying additional carboxyl groups, which further enhanced the cumulative effect of surface negative charges after hydroxyl modification. These results were consistent with the carboxyl content quantification shown in Figure 1. Furthermore, the contact angle analysis showed that the contact angle of unmodified PGs was large (61~72°). Whereas the contact angle of HM-PGs significantly decreased (38~45°) (*p* < 0.01, Figure 3C), suggesting that the hydrophilicity of HM-PGs was improved due to the successful introduction of carboxyl groups on the surface of PGs by TEMPO-mediated. These results demonstrated that the hydroxyl modification treatment of PGs by TEMPO could increase the carboxyl content on its surface, thereby significantly enhancing the electronegativity and hydrophilicity of HM-PGs.

### 3.4. Effect of HM-PG Treatment on the Adsorption Efficient of TTX

#### 3.4.1. The Influence of HM-PGs on the Removal Effect of TTX

**Figure 4:**
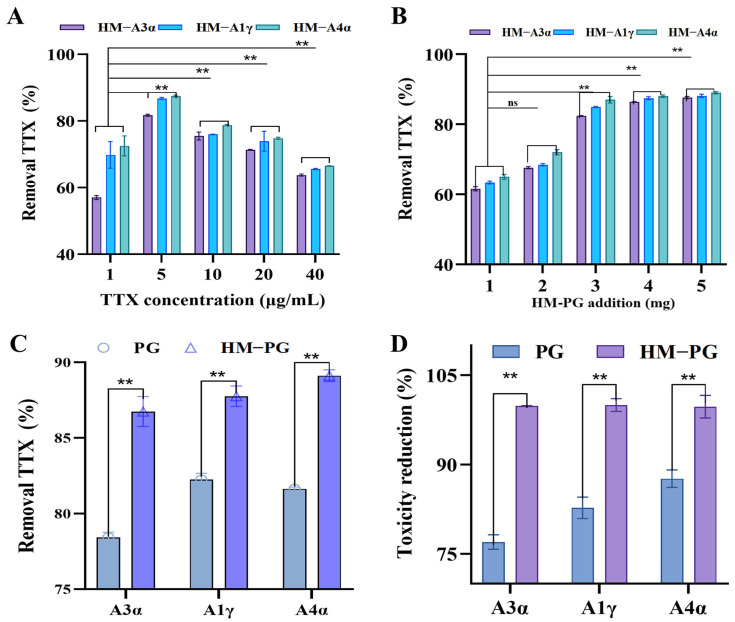
Effects of TTX concentration ((**A**)**,** n = 3) and HM-PG addition ((**B**)**,** n = 3) on TTX removal. Removal of TTX amount ((**C**)**,** n = 3) and toxicity ((**D**)**,** n = 6) by PGs before and after hydroxyl modification, under optimal conditions. All data are expressed as mean ± SD. A and B were analyzed for within-group significance, and C and D were analyzed for between-group significance; ns indicates not significant (*p* ≥ 0.05), ** indicates a statistically extremely significant difference (*p* < 0.01).

To evaluate the adsorption performance of the three HM-PGs towards TTX, the initial concentration of TTX and the HM-PGs dosage were optimized. It was found that the adsorption efficiency of three HM-PGs reached the highest value (81.75–86.76%) when the TTX concentration was 5 μg/mL (*p* < 0.01), suggesting that the ratio of the binding sites of TTX and HM-PGs was optimal at this concentration (*p* < 0.01, Figure 4A). Moreover, it was found that the adsorption efficiency leveled off when the amounts of HM-PGs added exceeded 3 mg, indicating that the adsorption system reached saturation (*p* < 0.01, Figure 4B). To validate the effect of hydroxyl modification on the adsorption capacity, the TTX removal efficiency of HM-PGs was compared with that of unmodified PG under the same conditions. As shown in Figure 4C, the removal rate of TTX by HM-PGs was significantly increased from 78.43% (PGs) to 89.12% (*p* < 0.01). Among them, HM-A4α exhibited the highest TTX removal capacity, which consisted of its highest carboxyl group content (Figure 1). Therefore, the significant enhancement of the adsorption performance could be attributed to the modification treatment of hydroxyl groups, which increased the hydrophilicity of PGs and the carboxyl density on the surface, thereby enhancing the adsorption interactions. In our previous study, we found that the adsorption capacity of PGs to TTX was 0.36 μg/mg [20]. However, in this study, the adsorption capacity of HM-PGs prepared by the TEMPO oxidation method was as high as 1.26 μg/mg, increasing by approximately 3.5 times [20]. Similarly, Mota et al. found that TEMPO oxidation treatment of cellulose hydrogels increased their carboxyl density and negative charge quantity, thereby enhancing their adsorption capacity for the cationic dye methylene blue [40]. It was consistent with our results, confirming that the TEMPO-mediated modification treatment could improve the adsorption performance of materials by introducing carboxyl groups. Furthermore, Alkassar et al. [9] found that 50 mg of cyclodextrin polymer was consumed when removing 80 ng of TTX from oyster tissues, and its efficiency was much lower than that of HM-PGs in this study. It can be seen that TEMPO oxidation can be used as an effective surface functional group modulation strategy to improve the adsorption efficiency of adsorbents.

Given that a reduction in TTX concentration may correspond to reduced toxicity, in vivo experiments were conducted to evaluate the toxicological impact of TTX following treatment with PGs before and after hydroxyl modification. As shown in Figure 4D, compared with the PGs, the toxicity of TTX after treatment with three HM-PGs significantly decreased by 77.01% to 99.85% (*p* < 0.01), which was consistent with the change of removal TTX, confirming that HM-PGs enhanced the adsorption effect of TTX.

#### 3.4.2. The Reduction Effect of HM-PGs on the Neurotoxicity of TTX

**Figure 5:**
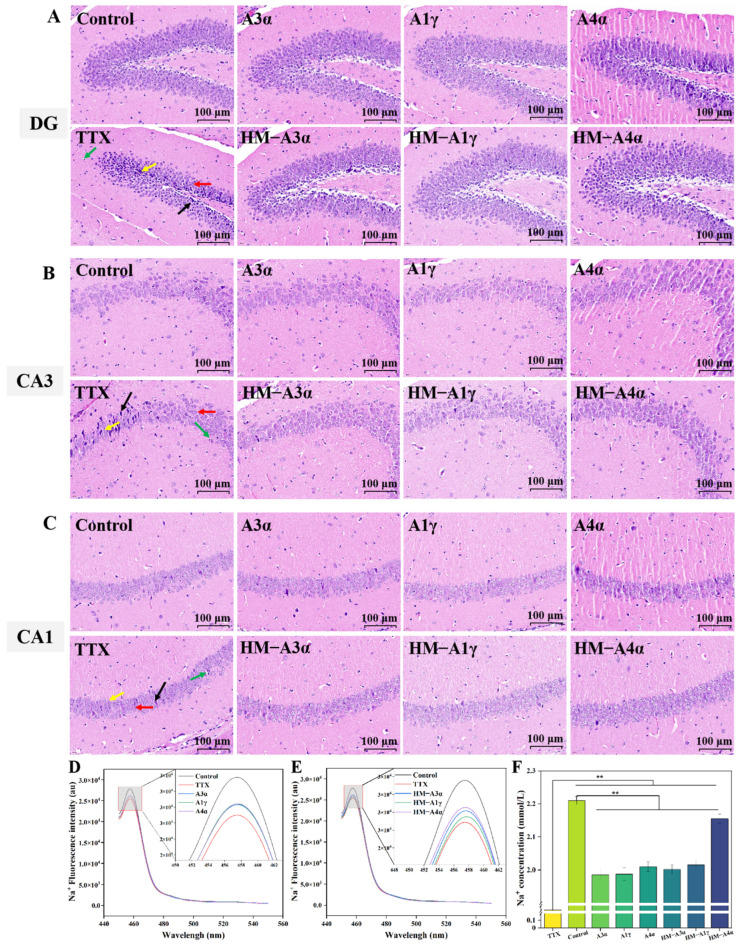
Pathomorphological changes of hippocampal DG area (**A**), CA3 (**B**) and CA1 (**C**) in mice (40×). The intracellular Na^+^ fluorescence (**D**,**E**) and concentration (**F**) in SH-SY5Y. Control: mice were injected intraperitoneally with only 1 mL of saline. All data are expressed as mean ± SD (n = 3). Significance between groups was analyzed; ** indicates a statistically extremely significant difference (*p* < 0.01).

As a neurotoxin, TTX mainly acts on the nervous system. The hippocampus, as a core region of neural function regulation, exhibits neuronal damage under TTX injury, which was a key target to study neurotoxicity [40,41]. To evaluate the reduction effect of HM-PGs on the neurotoxicity of TTX, hippocampal histopathology was examined in mice treated with TTX solutions before and after adsorption by HM-PGs. As shown in Figure 5A, in the control group, the neurons in the dentate gyrus (DG) region exhibited normal morphology, with densely packed cells, clearly visible nuclei and uniform cytoplasm. In contrast, the TTX group displayed pronounced pathological changes, including a reduction in neuronal density, deeply stained cells (yellow arrowheads), nuclear fragmented (black arrowheads), enlarged intercellular space (red arrowheads) and apoptosis (green arrowheads) (Figure 5A). Similarly, Buckmaster et al. showed that a certain degree of neuronal loss in the hippocampus was observed after TTX injection, consistent with our study [41,42]. Compared with the TTX group, neuronal damage was reduced in the PG and HM-PG groups, which had a higher density of neurons in the DG area, only mild perinuclear degeneration and no obvious necrosis or inflammatory infiltration. Notably, the HM-PG-treated groups showed greater protection than the unmodified PGs. Among them, HM-A4α has the most significant reduction effect on neurotoxic damage. This might be due to its superior adsorption capacity, which can effectively reduce the concentration of TTX, thereby reducing its damage to the hippocampus. Similar neuroprotective effects were also observed in the CA3 (Figure 5B) and CA1 (Figure 5C) regions of mice. In the TTX group, local neuronal necrosis (red arrow), decreased synaptic density (yellow arrow) and the abnormal increase of axonal fragmentation (black arrow) were observed, which was consistent with the decreased cell activity in the CA3 region found by Aranda et al. [42,43] after TTX injection. After adsorption treatment with PGs and HM-PGs, the neuronal damage in the CA3 and CA1 was alleviated. These results indicated that the reduction effect of HM-PGs was better than that of PGs, which was consistent with the results of DG.

In addition, studies have shown that TTX could specifically bind to the Nav1.1, Nav1.2, Nav1.6 and Nav1.7 subtypes of VGSC, thereby blocking Na⁺ inward flow [43,44]. SH-SY5Y cells have been proven to express Nav1.2 and Nav1.7, making them a suitable in vitro model for studying the neurotoxicity of TTX [44,45]. To study the effect of TTX on VGSC function, we used the sodium fluorescent probe SBFI-AM to monitor intracellular Na⁺ concentration as an indicator of VGSC activity. The results showed that the control group had strong intracellular Na⁺ fluorescence signals (Figure 5D), indicating normal VGSC activity and homeostasis intracellular Na⁺ level. In contrast, the Na⁺ fluorescence signals in the TTX group were significantly decreased, suggesting that TTX effectively blocked the VGSC and inhibited Na⁺ influx. Ngum et al. demonstrated that 22.3 nM of TTX completely blocked the entry of extracellular Na^+^ into the cell, similar to our study [45,46]. Following the adsorption of TTX by PGs (Figure 5D) and HM-PGs (Figure 5E), an increase in Na⁺ fluorescence intensity was observed in SH-SY5Y cells, suggesting that the residual TTX decreased due to its adsorption. Among them, a significant increase in Na⁺ fluorescence intensity was observed in the HM-A4α group, which is higher than other HM-PG groups. Correspondingly, the Na⁺ concentration of HM-PGs was higher than those of PGs, and the highest concentration was found in HM-A4α, reaching about 90.91% of the normal control group (*p* < 0.01, Figure 5F). These results suggested that the TTX after treatment with HM-PGs had lower neurotoxicity due to their better adsorption capacities to TTX than PGs.

### 3.5. Effect of Food Matrix on the Adsorption Capacity of TTX by HM-PGs

**Figure 6:**
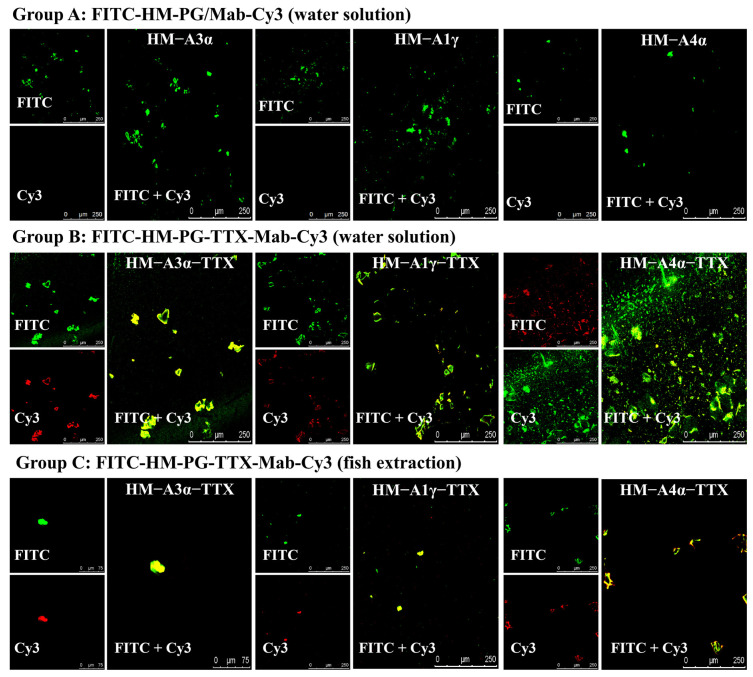
CLSM imaging visualization between three HM-PGs and TTX. (**Group A**): FITC-HM-PG/Mab-Cy3 control group; (**Group B**): FITC-HM-PG-TTX-Mab-Cy3 experimental group (water solution); (**Group C**): FITC-HM-PG-TTX-Mab-Cy3 experimental group (fish extraction).

To evaluate whether the food matrix interfered with the adsorption between HM-PGs and TTX, we performed a visualization analysis by CLSM. HM-PGs showed green fluorescence by FITC labeling, while TTX showed red fluorescence by binding Cy3 with the specific monoclonal antibody mAb. As shown in Figure 6, Group A, HM-PGs showed dispersed green fluorescent fragments, indicating that HM-PGs were successfully labeled by FITC and did not bind non-specifically to Cy3. In addition, the fragmented morphology of HM-PGs suggested partially depolymerized during hydroxyl modification. After the addition of TTX, a distinct red fluorescent signal was observed (Figure 6, Group B and C), which indicated that Cy3 successfully labeled TTX. However, red fluorescence was only observed in the presence of the specific mAb, further verifying that Cy3 could specifically label TTX. More importantly, obvious yellow fluorescence was observed under dual fluorescence channels, both in water and fish extract solution, indicating that the food matrix did not significantly interfere with the binding behavior of HM-PGs to TTX. Notably, the fluorescence intensity of the HM-A4α-TTX complex was observed to be higher than that of the HM-A3α-TTX and HM-A1γ-TTX complex. These results suggested that there was a strong adsorption interaction between HM-PGs and TTX and that the HM-A4α shows a higher binding capacity, which further supports its potential application in TTX removal. This may be due to the higher carboxyl group content of HM-A4α, which promotes hydrogen bonding and electrostatic interactions, thus increasing its binding capacity to TTX.

### 3.6. Analysis of Binding Mechanism Between HM-PGs and TTX

#### 3.6.1. Prediction of Binding Interaction and Mode Between HM-PG and TTX Based on Molecular Docking

**Figure 7:**
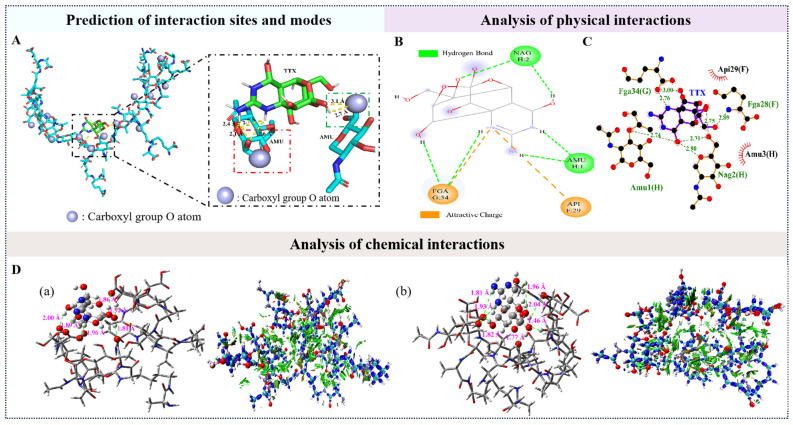
Schematic diagrams of the prediction of action sites (**A**), physical interaction (**B**,**C**) and chemical interaction ((**D**), (a) mode 1, (b) mode 2) between HM-PG and TTX.

To investigate the binding interaction and mode of HM-PG and TTX, the interaction sites and force types were analyzed by molecular simulation. First, the HM-PG structure was constructed based on the PG structure (PDB ID: 2MTZ). The Hydroxyl group in the PG sugar molecule was selectively converted into a carboxyl group using GaussView, with the modified sites highlighted as a purple sphere (Figure 7A), and the energy minimization was optimized by applying the Merck molecular force field (MMFF) in Chem3D to obtain a stable structure. Molecular docking between HM-PG and TTX was conducted through AutoDock Vina, which generated a stable HM-PG-TTX complex with a binding energy of −6.38 kcal/mol, indicating stable interactions. The interaction sites and functional groups were analyzed by PyMOL. The results showed that TTX could be stably embedded in the HM-PG backbone and formed multiple key interaction sites with HM-PG (Figure 7A). Among them, TTX formed hydrogen bonds with the original hydroxyl oxygen atom (red box) and the newly introduced carboxyl oxygen atom (green box), indicating that the introduction of the carboxyl group did not interfere with the binding of TTX, but enhanced the binding ability to TTX.

Furthermore, the interaction forces of the complex were analyzed by combining DS and LigPlot+. DS analysis showed that there were electrostatic and hydrogen bonding interactions between HM-PG and TTX (Figure 7B). The reason for this may be that hydroxyl modification introduces carboxyl groups on the surface of HM-PG, which significantly enhances the electronegativity of HM-PG and thus increases the electrostatic attraction with TTX guanidinium groups. Yu et al. demonstrated that the hydroxyl modification increased the carboxyl content of cellulose, which enhanced the electrostatic interaction between cellulose and Cd (II) and raised the adsorption capacity to 5.83 mg/g [46,47]. This was consistent with the results of the present study, indicating that the carboxyl group introduction could significantly enhance the adsorption capacity. In addition, the introduced carboxyl groups may promote the formation of a hydrogen bonding network between HM-PG and TTX guanidino and hydroxyl groups. The adsorption strength of methylene blue by the novel cellulose carboxylic acid adsorbent prepared by Chen et al. through TEMPO modification was significantly increased, mainly attributed to the synergy of hydrogen bonds and electrostatic interaction [47,48], which consistent our findings. LigPlot+ analysis further verified the multiple hydrogen bonds and revealed hydrophobic interaction between HM-PG and TTX (Figure 7C). It may be attributed to the hydroxyl modification that triggered the degradation of the partial structure of HM-PG, exposing more internal hydrophobic regions, and thereby promoting the hydrophobic interaction with TTX. In conclusion, hydroxyl modification effectively optimizes the electronegativity, hydrogen bond network and hydrophobic interface of HM-PG, enabling HM-PG to combine with TTX through physical actions including electrostatic, hydrogen bond and hydrophobic interaction.

More accurate binding modes of HM-PG to TTX were obtained by quantum chemical calculations as well as MD simulations. After HM-PG and TTX molecules were subjected to both Xtb optimization and MD, the two binding modes ((a) and (b)) with tight connections were screened (Figure 7D). It can be seen that HM-PG forms a cavity structure around the TTX molecule with (a) and (b) forming 6 and 7 hydrogen bonds (Figure 7D), which could be attributed to the carboxyl groups introduced by hydroxyl modification to promote the hydrogen bonding between HM-PG and TTX. Notably, in mode (b), a strong hydrogen bond with a bond length of 1.46 Å was observed. The Mayer bond order of 0.1229 calculated by Multiwfn (version 3.7)p roved the presence of chemical interaction [48,49]. In addition, the interaction strength visualization by VMD (Figure 7D) showed that hydrogen bonding networks with different strengths were observed in the blue-green region, further validating the physicochemical synergy. In conclusion, the adsorption of TTX by HM-PG showed not only physical interactions such as hydrophobicity and electrostatic forces, but also chemical interactions such as strong hydrogen bonding.

#### 3.6.2. Validation of Binding Forces Between HM-PGs and TTX

**Figure 8:**
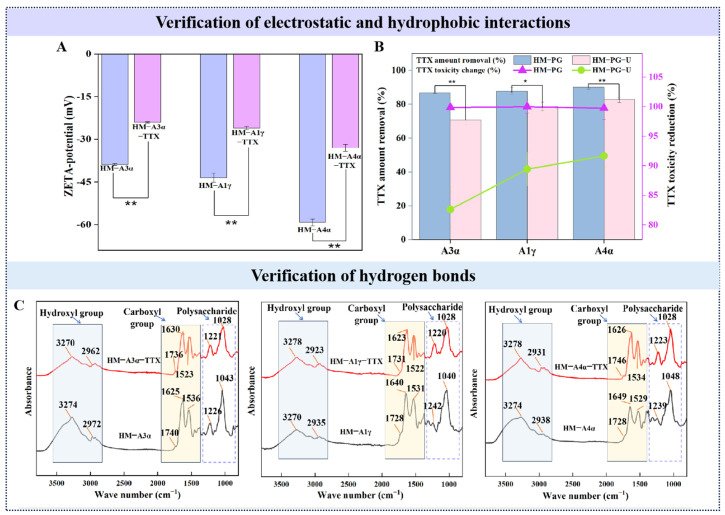
Validation of binding forces between HM-PGs and TTX. Zeta potential of HM-PGs and HM-PG-TTX ((**A**), n = 3). The amount and toxicity reduction effect of TTX by HM-PG before and after urea treatment ((**B**), n = 6); FTIR of HM-PG before and after adsorption of TTX ((**C**), n = 3). All data are expressed as mean ± SD. Significance between groups was analyzed; * indicates a statistically significant difference (*p* < 0.05), ** indicates a statistically extremely significant difference (*p* < 0.01).

The multiple interaction forces between HM-PG and TTX were verified through Zeta potential measurement, urea treatment experiments and FTIR spectroscopy. Firstly, the electrostatic interaction between HM-PGs and TTX was verified by measuring the Zeta potential change before and after TTX adsorption. Obviously, the Zeta potential of HM-PGs increased significantly from −36 mV to −25 mV after adsorption of TTX (*p* < 0.01, Figure 8A). This was probably caused by the electrostatic interaction between the positively charged guanidine in the TTX molecule and the carboxyl group on the surface of HM-PGs, which partially neutralized the negative charge of HM-PGs. The most significant change in the Zeta potential was observed in HM-A4α. It increased from −59 mV to −33 mV, and the corresponding TTX adsorption capacity was also higher (Figure 4C), which further demonstrated that electrostatic interactions played an important role in the adsorption of TTX. Amen et al. found that TEMPO-mediated oxidation could introduce abundant carboxyl groups on the nanocellulose, thereby enhancing the electrostatic adsorption of oxytetracycline and chloramphenicol, which was consistent with our study [49,50]. Similarly, Liu et al. observed a comparable charge neutralization trend in the adsorption of Cu(II) ions by TEMPO-oxidized cellulose, further affirming the importance of carboxyl functionality in facilitating electrostatic interactions [50,51], which is consistent with the present study. As a result, the TEMPO-mediated hydroxyl modification enhanced the electrostatic interaction between HM-PGs and TTX by increasing the carboxyl group content.

Studies have shown that the adsorption of toxins and organic pollutants by PG was usually driven by the synergistic effect of multiple intermolecular forces, including hydrophobic interaction, hydrogen bonds and electrostatic interactions. For example, Shen et al. found that hydrophobic interactions had a significant impact on the adsorption of acrylamide by PG [51,52]. In this study, the hydrophobic interaction between HM-PGs and TTX was verified using urea treatment of HM-PGs. Urea is a strong hydrogen bond donor and acceptor, which can form hydrogen bonds with functional groups such as carboxyl and amino groups of HM-PGs, thereby destroying the structure of its hydrophobic region. It was found that after urea treatment, the TTX removal rate of three HM-PGs decreased from 86.74% to 70.59% (*p* < 0.05, Figure 8B), indicating that hydrophobic interactions played an essential role in the adsorption process. In addition, the TTX toxicity reduction effect decreased from 99.83% to 81.61%, further proving that hydrophobic action plays an important role in the adsorption process. It was notable that HM-A3α showed the lowest TTX removal rate after urea treatment, probably due to the fact that HM-A3α contained more hydrophobic alanine and was more affected by urea. However, HM-A4α maintained a higher TTX removal rate due to its peptide bridge containing hydrophilic carboxyl groups provided by Asp, which was less affected by urea. Goyal et al. also found that PG has a higher affinity for the hydrophobic di(2-ethylhexyl) phthalate, and the adsorption capacity was significantly reduced by urea disrupting the hydrophobic region [52,53], which was consistent with our research results. Notably, the adsorption capacity of HM-PGs was decreased after urea treatment but still retained part of the adsorption effect, indicating that there might be other interactions besides the hydrophobic interaction.

In addition to electrostatic and hydrophobic interactions, we found that there was also a hydrogen bond between HM-PGs and TTX. As shown in Figure 8C, after adsorbing TTX, the C=O absorption peak of HM-PGs shifted from 1740 cm^−1^ to 1736 cm^−1^, which might be due to the participation of carboxyl groups in the formation of hydrogen bonds. A similar red-shifted was observed in the adsorption of oxytetracycline and chloramphenicol by TEMPO-modified cellulose nanofibers [49,50]. Meanwhile, the vibrational peaks of O-H and N-H were shifted from 3274 cm^−1^ and 1529 cm^−1^ to 3278 cm^−1^ and 1534 cm^−1^, respectively, which further verified that the hydrogen bond network was formed between the amine and hydroxyl groups in the TTX molecule and the carboxyl and amino groups of HM-PGs. Combining the molecular simulation and physicochemical results, it can be seen that the adsorption of HM-PGs with TTX relies not only on electrostatic interaction, but also on hydrogen bonding and hydrophobic interaction, forming a multi-mechanism synergistic mode. In summary, the adsorption of TTX by HM-PGs shows a synergistic adsorption effect of physical (electrostatic, hydrophobic and weak hydrogen bonds) and chemical (strong hydrogen bonds and Mayer bond order), significantly improving the adsorption efficiency of TTX and providing theoretical support for the optimal design of biological detoxification materials.

## 4. Conclusions

This modification increased the carboxyl group density of PGs by 68.42% and the surface electronegativity by 63.89%, resulting in about a 1.2-fold increase in TTX adsorption efficiency and effectively reducing its neurotoxicity. Compared with emerging adsorbents such as cyclodextrins and nerve cell membrane nanosponges, the HM-PGs developed in this study exhibited superior adsorption capacity of TTX, which provided the theoretical basis for the development of efficient and safe biological toxin adsorbents.

## Data Availability

The original contributions presented in this study are included in the article. Further inquiries can be directed to the corresponding author.

## Abbreviations

TTX	Tetrodotoxin
PG	Peptidoglycan
LAB	Lactic acid bacteria
VGSCs	Voltage-gated sodium channels
HM-PGs	Hydroxyl-modified PGs
SEM-EDS	Scanning electron microscopy energy dispersive spectroscopy
AFM	Atomic force microscopy
